# Breast weight and hormone receptor status in women with breast cancer

**DOI:** 10.1186/1477-7800-2-11

**Published:** 2005-05-16

**Authors:** M Salhab, W Al Sarakbi, K Mokbel

**Affiliations:** 1 St George's and The Princess Grace Hospitals, London, United Kingdom

**Keywords:** Breast cancer, postmenopausal, receptors, breast weight and aromatase

## Abstract

### Introduction

Aromatase activity in peripheral tissues including the breast is the main source of estrogen in postmenopausal women. There is evidence that local estrogen synthesis by breast aromatase contributes to mammary carcinogenesis. Therefore, we have postulated that high breast weight is associated with ER+ tumours.

## Patients and methods

The mastectomy specimen weight, ER and PgR status for 62 consecutive patients who had a total mastectomy for operable breast cancer were retrospectively reviewed. The ER/PgR positivity was assessed using immunohistochemistry (Quickscore system 0–8) by a breast pathologist. ER/PgR status was considered positive if the score was 4 – 8.

## Results

Overall the breast weight was higher in patients with ER+ disease. The weight was found to be significantly higher in women aged 50 years or older with ER+ tumours (669 vs. 220 grams, p = 0.015). There was no significant difference in breast weight between ER+ and ER- tumours in women aged less than 50 years (median weight: 440 vs.408 grams, p = 0.379). We observed a non-significant association between higher breast weight and PgR positivity (809 vs. 510 grams, p = 0.084) and absence of c-erb B2 (p = 0.088).

## Conclusion

In women aged 50 years or older with breast cancer, high breast weight is significantly associated with ER+ tumours. If this is confirmed in larger prospective studies, our findings may have implications regarding breast cancer prevention with anti-estrogens.

## Introduction

Breast cancer remains the most common cancer in females. It has been estimated that one in eight women will develop breast cancer during their lifetime in the USA [1]

Approximately two thirds of postmenopausal breast cancer patients have hormone dependent breast cancer that requires estrogen for tumour growth. It is well established that estrogens enhance growth and proliferation of certain target cells such as breast epithelial cells and estrogen dependent mammary carcinoma cells [2].

In postmenopausal women, estradiol does not appear to function as a circulating hormone; it is biosynthesized from androgens by the cytochrome P450 enzyme complex called aromatase [3] which is a product of the CYP19 gene, with the highest levels of this enzyme present in the peripheral adipose tissues of postmenopausal women [3]. Estrogen acts mainly at a local level as a paracrine or intracrine factor.

Aromatase has been found and measured in the stromal cell component of the normal breast as well as the breast tumour. Also, the enzyme has been detected in the breast epithelial cells in vitro [4-8]. Furthermore, expression of aromatase is highest in or near breast tumour sites [5,6,9,10]. It has been observed that the aromatase activity and expression is highest in the breast quadrant containing the tumour, such expression in the tumour containing quadrant is equal to that in the tumour itself, but double that in a quadrant of the same breast which does not contain a tumour which in turn is double the expression in the cancer free breast [10].

Evidence that postmenopausal obesity and weight gain are positively associated with postmenopausal breast cancer risk has been substantiated especially in women who never used hormone replacement therapy [11].

The aim of this study was to examine the hypothesis questioning the relationship between oestrogen and progesterone receptor status and breast tissue weight in postmenopausal women who had mastectomy for operable breast cancer.

## Patients and methods

The mastectomy specimen weight, estrogen receptor (ER) and progesterone receptor (PgR) status for 62 consecutive patients who had a total mastectomy for operable breast cancer were retrospectively reviewed. Breast specimen weight was obtained from the pathology laboratory reports in grams. ER and PgR status was determined by immunohistochemistry (IHC) using the Quickscore system by a breast pathologist. ER/PgR status was considered positive if the score was 4 – 8. We also examined other parameters including tumour size, grade, nodal status, c-erb B2 expression and patient's age.

Correlation between breast weight and receptor status was examined in two groups of patients according to their age, 50 years and older and younger than 50 years.

## Results

59 patients had invasive breast cancer and 3 patients had ductal carcinoma in situ (DCIS). Estrogen receptors were positive (ER+) in 50 patients (Quickscore: 4–8) and negative (ER-) in 12 patients (Quickscore: 0)

In general, breast weight was higher in patients with ER+ disease; median weight was 570 grams in ER+ patients compared to 413 grams in ER- patients (p = 0.04)

Patients were divided into two groups according to age. The first group consisted of patient aged 50 years and over (n = 43) with a median age of 60 years (range 51–83) and the second group contained those patients aged less than 50 years (n = 19) with a median age of 42 years (range 29–48).

**Figure 1:**
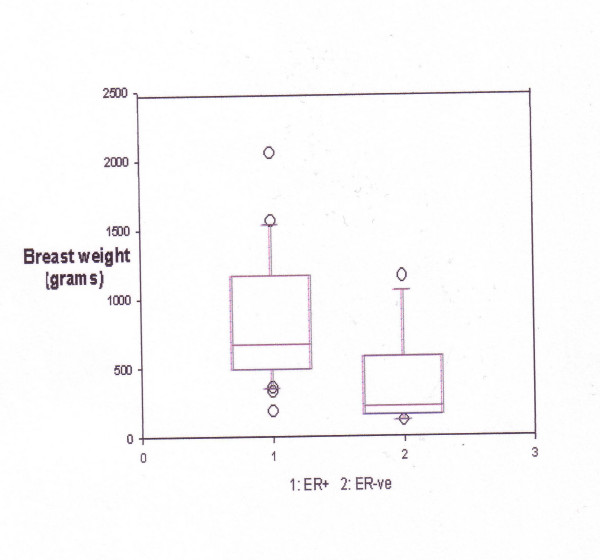
**ER status and mastectomy weight**. (Age = or > 50 years, p = 0.015)

Within the first group, breast weight was found to be significantly higher in women with ER+ tumours (669 vs. 220 grams, p = 0.015). Figure (1) demonstrates the relationship between the mastectomy specimen weight and ER status in the first group. Furthermore, when ER- and PgR – tumours were considered together the association was stronger (p = 0.003). On the other hand, there was no significant difference in breast weight between ER+ and ER- tumours in women in the second group (median weight: 440 vs.408 grams, p = 0.379).

There was no significant difference in tumour's size between ER+ and ER- patients. Furthermore, the tumour's grade was higher in ER- patients (p = 0.009) and patient's age had no significant impact on the receptor status.

We observed a non-significant association between higher breast weight and PgR positivity (809 vs. 510 grams, p = 0.084) and absence of c-erb B2 (p = 0.088).

Finally, there was no association between breast weight and nodal status or tumour's grade.

## Discussion

It has been established that obesity and weight gain are risk factors of breast cancer in postmenopausal women [12]. This relationship is explained by the fact that increased body weight is due to increased amount of adipose tissue in the body including breasts and subsequently increased of aromatase activity, thus increasing the local production of estrogen by aromatization of circulating androgens [13-16]. This process is a very important growth stimulating system in hormone dependent breast cancer [17].

Breast tissue and particularly the breast adipose tissue in postmenopausal women with breast cancer seem to have an increased aromatase expression. Agrawel et al studied 9 women undergoing breast reduction mammoplasty and 18 breast cancer patients undergoing mastectomies. Non-tumour bearing adipose samples from mastectomies expressed significantly more aromatase than adipose tissue taken from mammoplasty patients, a difference that is unlikely to be due the tumour's influence on aromatase expression [10].

The relationship between a higher body mass index (BMI) and the risk of ER+/PgR+ breast cancer has been studied previously [18-23]. In a case control study by Shelley et al a significantly increased risk of ER+/PgR+ breast cancer with increasing body size was observed. On contrast, other studies showed a greater frequency of ER- breast cancer among obese women [24-27].

The relationship between increased breast weight in postmenopausal women and receptor status has not been previously examined. In our study we hypothesised that in breast cancer patients; ER+ breast cancer was associated with a higher breast weight than ER- disease. This hypothesis was proved to be correct in postmenopausal women. Furthermore, such an association was not observed in women aged less than 50 years.

Our study has several limitations; firstly, we did not include other risk factors such as body mass index. Although a higher BMI is a recognised risk factor for ER+ disease, larger breasts are not always associated with a high BMI; higher breast weight could be found in a normal or low BMI postmenopausal women.

Secondly the family and HRT histories were not included in the analysis. Thirdly, the weight of the formatin-fixed mastectomy specimen was measured rather than the weight of the fresh specimen. However this applies to all specimens and is therefore unlikely to alter our findings. Finally we used the age of 50 years rather than the date of the last menstrual period to define the menopausal status. These limitations could be tackled in a larger prospective study.

In summary, in women aged 50 years or older with breast cancer, a higher breast weight seems to be significantly associated with ER+ disease. If this is confirmed in larger prospective studies, our findings may have implications regarding breast cancer prevention with anti-estrogens.

## References

[B1] Ries LAG, Kosary CL, Hankey BF, Miller BA, Edwards BK (1998). SEER Cancer Statistics Review, 1973–1995.

[B2] Dickson RB, Lippman ME, Mendelsohn J, Howley PH, Israel MA, Liotta LA (1995). Molecular basis of breast cancer. The Molecular Basis of Cancer.

[B3] Simpson ER, Mahendroo MS, Means GD, Kilgore MW, Hinshelwood MM, Graham-Lorence S, Amarneh B, Ito Y, Fisher CR, Michael MD (1994). Aromatase cytochrome P450, the enzyme responsible for estrogen biosynthesis. Endocr Rev.

[B4] James VH, McNeill JM, Lai LC, Newton CJ, Ghilchik MW, Reed MJ (1987). Aromatase activity in normal breast and breast tumor tissues: in vivo and in vitro studies. Steroids.

[B5] Bulun SE, Price TM, Aitken J, Mahendroo MS, Simpson ER (1993). A link between breast cancer and local estrogen biosynthesis suggested by quantification of breast adipose tissue aromatase cytochrome P450 transcripts using competitive polymerase chain reaction after reverse transcription. J Clin Endocrinol Metab.

[B6] Miller WR, Mullen P, Sourdaine P, Watson C, Dixon JM, Telford J (1997). Regulation of aromatase activity within the breast. J Steroid Biochem Mol Biol.

[B7] Reed MJ, Topping L, Coldham NG, Purohit A, Ghilchik MW, James VH (1993). Control of aromatase activity in breast cancer cells: the role of cytokines and growth factors. J Steroid Biochem Mol Biol.

[B8] Quinn AL, Burak WE, Brueggemeier RW (1999). Effects of matrix components on aromatase activity in breast stromal cells in culture. J Steroid Biochem Mol Biol.

[B9] Miller WR, O'Neill J (1987). The importance of local synthesis of estrogen within the breast. Steroids.

[B10] Agarwal VR, Bulun SE, Leitch M, Rohrich R, Simpson ER (1996). Use of alternative promoters to express the aromatase cytochrome P450 (CYP19) gene in breast adipose tissues of cancer-free and breast cancer patients. J Clin Endocrinol Metab.

[B11] Huang Z, Hankinson SE, Colditz GA, Stamper MJ, Hunter DJ, Manson JAE, Hennekens CH, Rosner B, Speizer FE, Willett WC (1997). Dual effects of weight and weight gain on breast cancer risk. Journal of the American Medical Association.

[B12] Yong LC, Brown CC, Schatzkin A, Schairer C (1996). Prospective study of relative weight and risk of breast cancer: the Breast Cancer Detection Demonstration Project follow-up study, 1979 to 1987–1989. Am J Epidemiol.

[B13] Cleland WH, Mendelson CR, Simpson ER (1985). Effects of aging and obesity on aromatase activity of human adipose cells. J Clin Endocrinol Metab.

[B14] Edman CD, Aiman EJ, Porter JC, MacDonald PC (1978). Identification of the estrogen product of extraglandular aromatization of plasma androstenedione. Am J Obstet Gynecol.

[B15] MacDonald PC, Edman CD, Hemsell DL, Porter JC, Siiteri PK (1978). Effect of obesity on conversion of plasma androstenedione to estrone in postmenopausal women with and without endometrial cancer. Am J Obstet Gynecol.

[B16] Carpenter CL, Ross RK, Paganini-Hill A, Bernstein L (1999). Lifetime exercise activity and breast cancer risk among postmenopausal women. Br J Cancer.

[B17] de Jong PC, Blankenstein MA, van de Ven J, Nortier JW, Blijham GH, Thijssen JH (2001). Importance of local aromatase activity in hormone-dependent breast cancer: a review. Breast.

[B18] Enger SM, Ross RK, Paganini-Hill A, Carpenter CL, Bernstein L (2000). Body size, physical activity, and breast cancer hormone receptor status results from two case-control studies. Cancer Epidemiol Biomarkers Prev.

[B19] Potter JD, Cerhan JR, Sellers TA, McGovern PG, Drinkard C, Kushi LR, Folsom AR (1995). Progesterone and estrogen receptors and mammary neoplasia in the Iowa Women's Health Study: how many kinds of breast cancer are there?. Cancer Epidemiol Biomarkers Prev.

[B20] Donegan WL, Johnstone MF, Biedrzycki L (1983). Obesity, estrogen production, and tumor estrogen receptors in women with carcinoma of the breast. Am J Clin Oncol.

[B21] Hislop TG, Coldman AJ, Elwood JM, Skippen DH, Kan L (1986). Relationship between risk factors for breast cancer and hormonal status. Int J Epidemiol.

[B22] Huang WY, Newman B, Millikan RC, Schell MJ, Hulka BS, Moorman PG (2000). Hormone-related factors and risk of breast cancer in relation to estrogen receptor and progesterone receptor status. Am J Epidemiol.

[B23] Sellers TA, Davis J, Cerhan JR, Vierkant RA, Olson JE, Pankratz VS (2002). Interaction of waist/hip ratio and family history on the risk of hormone receptor-defined breast cancer in a prospective study of postmenopausal women. Am J Epidemiol.

[B24] Papatestas AE, Panveliwalla D, Pertsemlidis D, Mulvihill M, Aufses AH (1980). Association between estrogen receptors and weight in women with breast cancer. J Surg Oncol.

[B25] McTiernan A, Thomas DB, Johnson LK, Roseman D (1986). Risk factors for estrogen receptor-rich and estrogen receptor-poor breast cancers. J Natl Cancer Inst.

[B26] Stanford JL, Szklo M, Boring CC, Brinton LA, Diamond EA, Greenberg RS (1987). A case-control study of breast cancer stratified by estrogen receptor status. Am J Epidemiol.

[B27] Yoo KY, Tajima K, Miura S, Takeuchi T, Hirose K, Risch H (1997). Breast cancer risk factors according to combined estrogen and progesterone receptor status a case-control analysis. Am J Epidemiol.

